# Substance Use Within Trials of Psychological Interventions for Psychosis: Sample Inclusion, Secondary Measures, and Intervention Effectiveness

**DOI:** 10.1093/schbul/sbae073

**Published:** 2024-05-23

**Authors:** Lauren Halsall, Anastasia Ushakova, Steven Jones, Samin Chowdhury, Laura Goodwin

**Affiliations:** Division of Health Research, Spectrum Centre for Mental Health Research, Lancaster University, Lancaster, England; Faculty of Health and Medicine, Centre for Health Informatics, Computing and Statistics, Lancaster University, Lancaster, England; Division of Health Research, Spectrum Centre for Mental Health Research, Lancaster University, Lancaster, England; Faculty of Health and Medicine, Lancaster Medical School, Lancaster University, Lancaster, England; Division of Health Research, Spectrum Centre for Mental Health Research, Lancaster University, Lancaster, England

**Keywords:** alcohol, psychosis, comorbidity, psychological interventions, substance

## Abstract

**Introduction:**

Current clinical guidelines recommend that patients with co-occurring psychosis and alcohol or substance use disorders (A/SUD) receive evidenced-based treatment for both disorders, including psychological intervention for psychosis. However, the efficacy of such treatments for individuals with co-occurring psychosis and A/SUD is unclear.

**Study Design:**

Randomized controlled trials (RCTs) of psychological interventions for psychosis were systematically reviewed, to investigate how alcohol and substance use has been accounted for across sample inclusion and secondary measures. Findings from trials including individuals with co-occurring alcohol or substance use issues were then narratively summarized using the Synthesis Without Meta-Analysis guidelines, to indicate the overall efficacy of psychological interventions for psychosis, for this comorbid population.

**Study Results:**

Across the 131 trials identified, 60.3% of trials excluded individuals with alcohol or substance use issues. Additionally, only 6.1% measured alcohol or substance use at baseline, while only 2.3% measured alcohol or substance use as a secondary outcome. Across trials explicitly including individuals with alcohol or substance use issues, insufficient evidence was available to conclude the efficacy of any individual psychological intervention. However, preliminary findings suggest that psychoeducation (PE) and metacognitive therapy (MCT) may be proposed for further investigation.

**Conclusion:**

Overall, co-occurring alcohol and substance use issues have been largely neglected across the recent RCTs of psychological interventions for psychosis; highlighting the challenges of making treatment decisions for these individuals using the current evidence base.

## Introduction

Over 50% of individuals diagnosed with a psychotic disorder are estimated to experience alcohol or substance use issues.^1–3^ Both clinically and socially, the addition of an alcohol or substance use disorder (A/SUD) associates with poorer outcomes for individuals with psychosis, including increased psychotic symptoms and mortality.^4–6^ Resultingly, the effective management of co-occurring psychosis and A/SUD is of crucial importance, and investigation into psychosis should account for A/SUD.

According to the National Institute for Health and Care Excellence (NICE) treatment guidelines,^7^ patients with co-occurring psychosis and A/SUD should be offered evidenced-based treatments for both disorders. Specifically, combined antipsychotic medication and psychological intervention is recommended for the management of psychosis, with cognitive behavioral therapy (CBT) and family therapy (FT) outlined as effective psychological interventions.^8^ Additionally, a recent meta-analysis concluded that, overall, cognitive remediation, social skills training, psychoeducation (PE), and mindfulness-based therapies, alongside the recommended CBT and FT, are also more efficacious that routine treatment for the alleviation of psychotic symptoms.^9^ Consequently, a range of psychological interventions may be considered alongside antipsychotic medication for the treatment of psychosis, including for individuals with co-occurring A/SUD.

However, delivering standard psychosis treatment to individuals with co-occurring A/SUD may not be appropriate, as individuals with co-occurring disorders are often excluded from clinical trials.^10^ Indeed, meta-analytical synthesis revealed that almost 70% of behavioral intervention trials published between 2000 and 2014 excluded individuals with multimorbid conditions.^11^ Furthermore, the most recent review of psychological interventions for psychosis excluded samples with co-occurring A/SUD,^9^ and therefore the overall efficacy of psychological interventions for psychosis for this comorbid population remains unclear. As treatment outcomes can be worsened by the presence of a comorbid disorder,^12,13^ findings regarding intervention effectiveness should not be uncritically generalized to excluded, comorbid populations. Resultingly, while systematic issues with the representativeness of clinical trial samples have been previously established,^11^ investigation into A/SUD exclusion for psychosis intervention trials is necessary, to understand the degree to which existing findings apply to individuals with co-occurring A/SUD. This is particularly relevant given the recommendations from clinical guidelines to routinely deliver psychological interventions for psychosis,^7^ and the high prevalence of comorbid A/SUD for individuals with psychosis.^1–3^ Beyond targeting psychosis, psychological interventions for psychosis may have increased treatment potential for individuals with co-occurring A/SUD if substance use is also targeted and reduced.^14^ Fully integrated treatment, within which both disorders are targeted,^15^ remains the gold standard for managing co-occurring mental health and substance abuse issues.^16,17^ However, nonintegrated interventions for psychosis may also have the potential to beneficially reduce substance use, as substance use is associated with psychotic symptom severity, and thus symptom improvement may conversely reduce substance use; as reported across interventions for other mental health conditions.^4,18^ However, substance-related outcomes across psychological interventions trials for psychosis have not been systematically explored.

Overall, whether substance use is accounted for across trials of psychological interventions for psychosis remains unclear, and the effectiveness of these interventions for individuals with co-occurring A/SUD remains unclear. However, clinical guidelines continue to recommend these interventions for delivery to individuals with co-occurring psychosis and A/SUD, and therefore it remains plausible that these individuals are receiving nonoptimal treatment. The current research therefore aims to investigate the following research questions (RQs):

(1) What proportion of randomized controlled trials (RCTs) of psychological interventions for psychosis exclude individuals with alcohol or substance use issues?
(2) What proportion of RCTs of psychological interventions for psychosis measured alcohol or substance use as a secondary outcome?
(3) When alcohol or substance use has been measured as a secondary outcome within RCTs of psychological interventions for psychosis, which measures have been used?
(4) Are psychological interventions for psychosis effective for individuals with co-occurring alcohol or substance use issues?

## Methods

A systematic review was conducted to investigate how alcohol and substance use has been accounted for across RCTs of psychological interventions for psychosis. The review was preregistered on PROSPERO, registration number: CRD42023396418, and reported in accordance with the Preferred Reporting Items for Systematic Reviews and Meta-Analyses (PRISMA) reporting guidelines.^19^

### Search Strategy

Titles, abstracts, and keywords across the electronic databases MEDLINE, Embase, PsycINFO, Scopus, and the Cochrane CENTRAL register, were searched on February 7, 2023 using the following search terms, combined using Boolean operators:

- Psychosis terms: psychosis, psychoses, psychotic*, schizo*, catatoni*, delusion*, hallucinat*.
- Psychological intervention terms: “psychological intervention*,” “cognitive remediation,” PE, “supportive therap*,” “cognitive therap*,” “behaviour* therap*,” “behavior* therap*,” CBT, “compassion focused therap*,” CFT, “acceptance and commitment therap*,” “mindful* therap*.”

Searches were restricted to reports available in English, with an additional limit set to identify RCTs where possible. Searches were also restricted to trials published from 2011 onwards following the publication of the NICE guidelines for co-occurring psychosis and substance misuse,^7^ which outlined that patients should not be excluded from mental health treatment due to their substance misuse. No further search limits were set. The primary reviewer performed 100% of the screening, while a second reviewer independently screened 10% of titles, abstracts, and full texts. Reasons for exclusion were recorded at the full-text stage, and discrepancies between reviewers were settled through discussion. Following formal screening, reference lists of eligible studies were hand searched to identify any further literature.

### Inclusion Criteria

Only RCTs, considered the “gold standard” for investigating intervention efficacy,^20^ were eligible for inclusion within the review. The remainder of the inclusion and exclusion criteria will be outlined using the PICO (population, intervention, comparator, and outcome) format.^21^

#### Population.

The population of interest was individuals diagnosed with any schizophrenia spectrum or other psychotic disorder, according to any recent diagnostic criteria. Samples with additional psychiatric comorbidities remained eligible for inclusion as it is estimated that over 50% of psychosis patients suffer from co-occurring mental health disorders,^22,23^ and therefore exclusion of such individuals would prevent generalizability of findings to a substantial proportion of the target population. Likewise, samples of special populations, such as incarcerated or homeless individuals, also remained eligible for inclusion, as these individuals are of greater A/SUD risk,^24,25^ and therefore an understanding of how substance use has been accounted for across trials of psychological interventions for psychosis within these populations is crucial. Additionally, samples with mixed diagnoses were eligible for inclusion if the majority of participants (>50%) were diagnosed with a psychotic disorder to prevent the exclusion of relevant literature, given the expectation that many interventions will have been delivered to mixed samples (eg, samples of individuals with any severe mental illness). Only adult samples (≥18 years) were eligible for inclusion.

#### Intervention.

Interventions of interest were any psychological interventions, defined as psychological theory-driven activities or therapies.^9^ Trials of all durations, delivery modes, and psychological approaches were eligible for inclusion. Additionally, trials including a pharmacological component were eligible for inclusion, as NICE guidelines for psychosis treatment recommend conjunctive psychological and pharmacological intervention,^8^ however, purely pharmacological trials were excluded. Any interventions primarily targeting psychosis at the disorder level or specifically targeting a psychotic disorder primary symptom (eg, interventions aimed at alleviating hallucinations) were eligible for inclusion within the review. However, interventions with alternative primary targets, such as trauma interventions or approaches targeting medication adherence, were excluded, even if the primary outcomes were measured. Likewise, interventions targeting both psychosis and A/SUD were excluded, as evaluating this treatment approach was not the focus of the current review.

#### Comparator.

To ensure that findings were clinically meaningful, the comparator of interest was treatment as usual (TAU). As routine psychiatric care for psychosis varies substantially across clinical practice and a range of approaches are currently recommended for treatment of psychosis,^8^ no attempts were made to standardize or strictly define eligible TAU. However, as current treatment guidelines explicitly state that psychosis patients should receive psychological care alongside pharmacological management,^8^ trials preventing participants within the control condition from accessing psychological care, or trials comparing psychological intervention to antipsychotic medication only, were excluded, even if this comparator was defined as TAU.

Additionally, NICE guidelines outline that supportive therapy (ST) may be considered for management of psychosis when appropriate for the service user,^8^ and therefore trials comparing psychological interventions to ST were also eligible for inclusion. All alternative comparator conditions were ineligible for inclusion, including trials comparing multiple types of psychological intervention, or comparing multiple versions of the same type of psychological intervention, as the purpose of the current review was not to establish superiority between psychological interventions, or to establish superior components of single psychological interventions.

#### Outcome.

The primary outcomes of interest were psychotic symptoms or psychiatric severity for psychosis. Any standardized measures, such as the Positive and Negative Syndrome Scale (PANSS) or Brief Psychotic Rating Scale (BPRS) for psychotic symptoms,^26,27^ or the Clinical Global Impressions Scale (CGI) for psychiatric severity,^28^ were eligible for inclusion. Only trials measuring at least 1 primary outcome were included within the review. Additional to the primary outcomes, the secondary outcomes of interest were substance use, quality of life, and general functioning, measured using any standardized measure, as well as any measure of relapse or hospitalization. Information on adverse effects or treatment failures, including treatment failures for outcomes that were neither primary nor secondary outcomes within the current review, was also of interest, to ensure that review conclusions consider any potential negative impacts of interventions on patients.

### Data Extraction

The Cochrane Handbook for Systematic Reviews of Interventions outlines a checklist of items to consider for extraction,^29^ which was used to develop a codebook for data extraction. From studies excluding participants with a co-occurring A/SUD, or from studies not recording participants alcohol or substance use within the report, only study details (authors and publication year) and substance-related exclusion criteria was extracted, to enable the investigation of RQs 1–3.

From trials that included individuals with alcohol or substance use issues and provided such description within the report, and therefore were included within the full review, participant information (age, gender distribution, diagnostic details, and sample size), intervention details (type, duration, and latest available follow-up), comparator details (TAU or ST), and primary and secondary outcome measures (measures used and findings reported) were extracted, to enable the investigation of RQ4. For all outcomes, data from the latest available follow-up was extracted. Psychological intervention type was coded using criteria adapted from McGlanaghy et al’s classification of psychological interventions,^9^ provided in the supplementary materials. If essential data were missing, authors were contacted and given a period of 1 month to respond, before the trial was excluded.

### Risk of Bias

Risk of bias was assessed for each trial included within the full review using the Cochrane Risk of Bias 2 Tool.^30^ Briefly, risk of bias was assessed across the following domains: randomization processes, deviation from the intended interventions, missing outcome data, outcome measurement, and result reporting, generating an overall risk of bias assessment (full assessment provided in the supplementary materials). Risk of bias will be explicitly discussed and evaluated in relation to trial findings, and therefore trials determined to be high risk of bias were not excluded from the review.

### Data Analysis

The proportion of trials excluding participants with alcohol or substance use issues was calculated, to investigate RQ1. Exclusion criteria were then quantitatively described, reporting the number of trials excluding individuals with alcohol or substance use issues across a range of severities (eg, abuse, dependence), timeframes (eg, current, lifetime), substances, and principalities (eg, primary diagnosis, any diagnosis). Additionally, the proportion of trials not measuring alcohol or substance use within the sample was calculated, as well as the proportion of trials measuring alcohol or substance use as a secondary outcome followed by the number of trials using each identified outcome measure, to investigate RQ2 and RQ3.

For investigation of RQ4, all analyses were restricted to trials explicitly including participants with alcohol or substance use issues. Findings of the remaining trials were synthesized to investigate the overall efficacy of psychological interventions for psychosis, for individuals with co-occurring alcohol or substance use issues. Across included trials, clinical, methodological, and statistical heterogeneity was substantial, with studies reporting a range of psychological interventions, samples, outcome measures, and measures of effect, and therefore meta-analytical synthesis was deemed inappropriate. Consequently, studies were narratively synthesized using the Synthesis Without Meta-Analysis (SWiM) framework; developed using formal consensus and expert consultation to support transparent and systematic narrative reporting of findings.^31^

Within SWiM analyses, findings were synthesized using vote counting with effect direction as the standardized metric; recommended by Cochrane collaborators when reported measures of effect are inconsistent between studies.^32^ In accordance with such guidance,^32^ the overall effect estimate for psychotic symptoms and psychiatric severity within each included trial was categorized as intervention improvement, intervention deterioration, or no clear effect. Importantly, as required by updated Cochrane guidelines for acceptability of narrative synthesis, both statistical significance and effect size were ignored in effect direction calculations due to the arbitrary nature of statistical significance and effect size thresholds and reliance upon statistical power for significance,^32^ although they remain reported within tabulated summaries of the included trials for clarity and completeness. In accordance with Boon and Thomson’s guidelines for applying the updated Cochrane guidance, overall effect direction within a trial reporting multiple outcomes within the same domain (eg, multiple subscales on psychotic symptom scales) was calculated using the following criteria: <70% of findings reporting consistent direction of effect = no clear effect, with ≥70% of findings report consistent direction of effect = report direction of effect.^32,33^ Notably, as updated guidance recommends ignoring statistical significance within effect direction narrative syntheses,^32,33^ trials reporting 1 single outcome within a domain could only report no effect if data between groups were identical. Consistent with Cochrane guidelines,^32^ the number of trials reporting each overall effect direction, for each outcome, was then calculated, to indicate the overall effect direction. Within analyses, trials are grouped by psychological intervention type.

As effect direction is appropriate for use across multiple measures of effect,^32^ studies reporting any measure of effect were included. However, where multiple measures of effect were available for the same outcome (eg, both change scores and endpoint analysis presented), measures of effect accounting for baseline scores were selected where possible to determine effect direction (eg, change scores, endpoint analysis controlling for baseline scores) to limit the confounding influence of baseline scores. No trial reported multiple measures of effect accounting for baseline scores with sufficient data for inclusion in the current study given that statistical significance was ignored, and therefore no decisions were made as to which of these measures of effect to extract superiorly.

To visually represent data, an effect direction plot was generated, separated within the plot by psychological intervention type. As outlined by Boon and Thomson, sample size was reflected using arrow size, with larger arrows used within the plot to depict larger samples, as according to effect direction plot guidelines.^33,34^ As with the SWiM analyses, statistical significance and effect size were ignored upon recommendation by Cochrane collaborators,^32^ and therefore no sign test or measure of effect magnitude was added to plots.

## Results

**Fig. 1.**
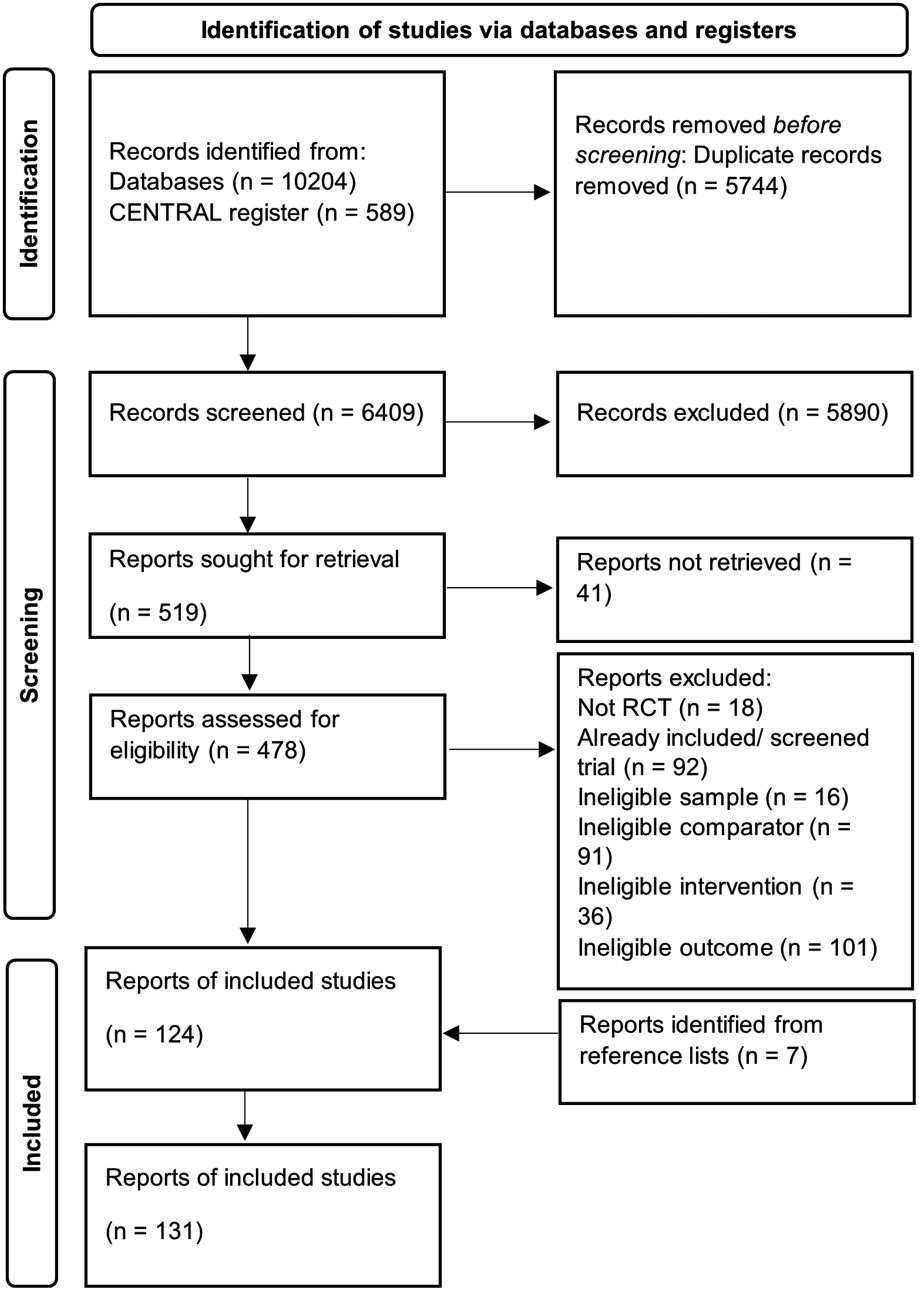
PRISMA flow diagram.^19^

The full screening process is displayed in the PRISMA diagram (figure 1).

Briefly, after the removal of duplicates, 6409 reports were identified from the database and register searches, with 519 full texts remaining for inclusion after screening of titles and abstracts. Across these 519 full texts, 124 trials of psychological interventions for psychosis were identified for inclusion within the review. Reference lists of these 124 trials were then hand searched, identifying another 7 trials for inclusion. Overall, therefore, 131 trials of psychological interventions for psychosis were identified (a list of all included studies is provided in the supplementary materials). Level of consideration of alcohol or substance use across the trials is outlined in table 1.

**Table 1.** Number of Trials Accounting for Alcohol or Substance Use Across Inclusion Criterion or Secondary Measures

Level of Consideration of Substance Use	Number of Trials (%)
Not measured/reported	44 (33.6)
Excluded participants with substance use issues*	79 (60.3)
Reported baseline substance use	8 (6.1)
Measured as secondary outcome	3 (2.3)

^a^Substance use issues include dependence, abuse, substance use disorder (SUD), and any further concerns.

As shown in table 1, a substantial number of reports did not measure alcohol or substance use across the sample, while only a small number of reports included measures of baseline alcohol or substance use. For ease of interpretation, the remaining results section will be structured into sections corresponding to the RQs.

RQ1: What proportion of RCTs of psychological interventions for psychosis exclude individuals with alcohol or substance use issues?

Across the 131 trials identified, 79 trials (60.3%) excluded participants based on alcohol or substance use status. A descriptive summary of exclusion criterion is displayed in table 2.

**Table 2.** The Number of Trials Reporting Each Alcohol or Substance-Related Exclusion Criteria

Exclusion Criterion	Number of Trials (%)
Severity-based definitions	
Dependence	25 (31.6)
Abuse	22 (27.8)
Dependence and abuse	10 (12.7)
A/SUD	10 (12.7)
Other	12 (15.2)
Timeframe-based definitions	
Current/active with specific timeframe	16 (20.3)
Current/active without specific timeframe	11 (13.9)
Lifetime/history	9 (11.4)
Unspecified	43 (54.4)
Substance-based definitions	
Substances	50 (63.3)
Substances and alcohol	25 (31.6)
Substances, but not nicotine/caffeine	1 (1.3)
Substances, but not nicotine/caffeine/cannabis	1 (1.3)
Other	2 (2.5)
Principality-based definitions	
Primary diagnoses only	16 (20.3)

A/SUD = alcohol or substance use disorder.

As shown in table 2, specific alcohol or substance use-related exclusion criterion varied substantially between trials. Most commonly, dependence was outlined as the criterion for exclusion, while timeframes of issues were most frequently unspecified.

RQ2 and RQ3: What proportion of RCTs of psychological interventions for psychosis measured alcohol or substance use as a secondary outcome, and which measures of alcohol or substance use are used?

Only 3 trials measured alcohol or substance use as a secondary outcome. Notably, 2 of these 3 trials also excluded individuals with alcohol or substance use issues.^35,36^ A description of the measured used is provided in table 3.

**Table 3.** The Number of Trials Measuring Alcohol or Substance Use Using Each Outcome Measure

Outcome Measure	Number of Trials
ASSIST	1^37^
AUDIT	2^36,37^
SDS	1^37^
DAST	1^36^
Assessed by case manager (criteria NR)	1^35^

ASSIST = Alcohol, Smoking, and Substance Involvement Screening Test; AUDIT = Alcohol Use Disorder Identification Test; SDS = Severity of Dependence Scale; DAST = Drug Abuse Screening Test; NR = not reported. Subscript indicates the trial within which the measure was included.

As shown in table 3, a variety of outcome measures were used to measure alcohol or substance use; most common of which was the AUDIT.

RQ4: When individuals with co-occurring alcohol or substance use issues are included in the sample, are psychological interventions for psychosis efficacious?

Seven trials of psychological interventions for psychosis explicitly included participants with alcohol or substance use issues. Across the 7 trials, the psychological interventions of PE, metacognitive therapy (MCT), CBT, and a specialized assertive early intervention program (OPUS) were investigated, in comparison to TAU (full study characteristics are provided in the supplementary material). An effect direction plot, grouped by psychological intervention type and then ordered by overall risk of bias, is presented in table 4 for all primary and secondary outcomes. As psychiatric severity and hospitalization were not measured within any trial, they were not included in the plot.

**Table 4.** Effect Direction Plot for Primary and Secondary Outcomes for Trials of Psychological Interventions for Psychosis, Including Individuals With Co-occurring Alcohol or Substance Use Issues

Citation	Psychotic Symptoms	Substance Use	Quality of Life	General Functioning	Relapse
PE					
Aho-Mustonen (2011)^38^	**▲**		**▼**		
MCT					
Favrod (2014)^39^	**▲**3				
Kuokkanen (2014)^40^	**▲**2				
Moritz (2011)^41^	**◄►**6		▲4		
CBT					
Gleeson (2013)^37^	**▼**8	**▲**4	**▲**4	**▼**	**▲**
Khazaal (2015)^42^	**◄►**5			**▲**2	
OPUS					
Secher (2015)^43^	◄►3			**◄►**2	

▲ = improvement; ◄► = no clear effect; ▼ = deterioration. Size of intervention group: ▲ = >250, ▲ = 50–250, ▲ = <50, as according to Boon and Thomson.^33^ The number of outcomes contributing toward the overall effect direction is reported next to arrow; where no number is reported, then *n* = 1. PE = psychoeducation; MCT = metacognitive training; CBT = cognitive behavioral therapy; OPUS = specialized assertive early intervention program.

### Psychotic Symptoms

As shown in table 4, an insufficient number of studies investigating any type of psychological intervention were available to indicate an overall effect direction. Preliminary findings may suggest a possible benefit of MCT, with 2 trials reporting overall treatment improvement and 1 reporting no clear effects, as well as a possible benefit of PE, with the 1 trial reporting overall treatment improvement, compared to the control group. No benefits were indicated for CBT or the OPUS intervention.

### Secondary Outcomes

Findings across secondary outcomes were limited. Only 1 trial (CBT) measured substance use and relapse, reporting intervention improvements, compared to the control group, across both outcome domains. Findings for general functioning were mixed across psychological intervention type, with 1 trial (CBT) reporting an intervention improvement, 1 trial (CBT) reporting an intervention deterioration, and 1 trial (OPUS) reporting no clear effect of the intervention, compared to the control group. Finally, findings for quality of life were also mixed across psychological intervention type, with 2 trials trial (CBT and MCT) reporting an intervention improvement and 1 trial (PE) reporting an intervention deterioration, compared to the control group. Overall, as with primary outcomes, insufficient evidence is available to produce overall findings.

### Other Adverse Events or Treatment Failures

Importantly, 2 trials reported treatment failures associated with intervention delivery, aside from the treatment deteriorations discussed above. One trial reported that, at follow-up, participants who had received PE reported greater increases in irritability than participants who had received TAU.^38^ Additionally, across the duration of another trial, participants receiving the OPUS intervention spent more days in a homeless shelter than participants receiving TAU.^43^ No additional adverse events or treatment failures were reported.

## Discussion

### Key Findings

Across the existing literature, 60.3% of RCTs of psychological interventions for psychosis excluded individuals with alcohol or substance use issues. This finding supports a growing body of reviews reporting that comorbid health conditions are often criteria for exclusion across intervention trials^11,44–46^ and is consistent with findings regarding the commonality of specific substance-related exclusion across PTSD treatment trials.^12^

Furthermore, alcohol or substance use was rarely reported within trials, with only 6.1% reporting sample baseline measures and therefore explicitly including individuals with alcohol or substance use issues, and only 2.3% measuring alcohol or substance use as a secondary outcome across 5 outcome measures. Although a novel finding across the psychosis literature, conclusions mirror those reported across trials of PTSD treatment; only 7.7% of which measured substance-related outcomes across trials conducted between 1980 and 2015.^12^

Overall, therefore, substance use is largely unaccounted for across trials of psychological interventions for psychosis. Given that over 50% of individuals diagnosed with a psychotic disorder are estimated to experience alcohol or substance use issues,^1–3^ findings indicate that current literature does not reflect the realities of the clinical population and raises concerns about bias. Consequently, the delivery of psychological interventions to comorbid populations, and therefore a substantial proportion of patients with psychosis, is challenged. This is particularly relevant as treatment outcomes can be worsened by the presence of a comorbid disorder.^12,13^ Where appropriate, future trials of psychological interventions for psychosis should include individuals with co-occurring A/SUD, and aim to measure alcohol or substance use at baseline and at follow-up, to ensure that findings are applicable to the wider clinical population and can be generalized appropriately.

As the existing literature is not representative of the clinical population, there is a need for evidenced-based conclusions regarding the efficacy of psychological interventions for psychosis for individuals with co-occurring A/SUD. The current review found insufficient evidence to conclude superior efficacy of any specific psychological intervention for individuals with co-occurring A/SUD, compared to TAU. However, preliminary evidence proposes PE and MCT as possible targets for future investigation.

### Strengths and Limitations

The review comprehensively followed both SWiM guidelines^31^ and the PRISMA reporting guidelines^19^; enabling robust and systematic findings and reporting in the absence of meta-analytical processes. Likewise, conclusions regarding intervention efficacy are strengthened by their basis on the standardized metric of effect direction; bypassing the challenges of traditional significance thresholds.^32^

Nevertheless, potential limitations of the review should be considered critically, and primarily lie within the limitations of the included evidence body. Notably, the number of trials included within the full review is limited, with no intervention type investigated in more than 3 studies and many types not included at all; including FT, as explicitly recommended by NICE for the treatment of psychosis.^8^ Furthermore, sample sizes of the included trials are notably small, with only 2 of the included trials reporting sufficient statistical power.^42,43^ Within the trials themselves, the primary outcome of psychotic severity was not measured within any trial. As psychiatric severity provides a broader disorder picture than symptomatology alone, with measures typically encompassing wider behavior, functioning, and impact,^28^ the primary focus on psychotic symptoms alone within the current review may prevent the discovery of potential wider treatment effects. Similarly, secondary outcome measurement of substance use, quality of life, general functioning, relapse, and hospitalization, was notably limited across trials, despite outcomes being qualitatively described by patients as central to their recovery.^47–49^ Broadly, more high-quality research into the efficacy of psychological interventions for psychosis with carefully selected outcomes, for individuals with co-occurring alcohol or substance use issues, is necessary, to expand and strengthen current understanding.

Beyond concerns with review scope, inadequate reporting and limited analyses is of notable concern. Critically, 33.6% of trials did not exclude individuals with alcohol or substance use issues, but also did not report alcohol or substance use within the sample. As comorbidity of psychosis and A/SUD is estimated to be over 50%,^1^ many of these trials likely included individuals with alcohol or substance use issues, however, they could not be included within the full review. Alternatively, no trial included within the full review reported subgroup analyses based on alcohol or substance use, and therefore overall estimates were instead extracted, despite inclusion of participants without alcohol or substance use issues. Future studies should consider measuring and explicitly reporting baseline alcohol or substance use when investigating individuals with psychotic disorders, to enable a clear and comprehensive understanding of the sample being investigated, and thus allow findings to be appropriately situated. Furthermore, although the appropriateness of subgroup analysis should be carefully considered for each individual trial, future similar studies may consider stratifying analyses by alcohol or substance use to enable conclusions specifically regarding the efficacy of psychological interventions for psychosis for individuals with co-occurring A/SUD to be drawn.

### Conclusions

Overall, the current review exposes a wide gap in the existing literature by highlighting that alcohol and substance use is not currently accounted for across trials of psychological interventions for psychosis. As such, evidence-based conclusions regarding the efficacy of psychological interventions for psychosis cannot be drawn for individuals with co-occurring A/SUD, despite recommendation by clinical guidelines to deliver such interventions to this population. Future research should include individuals with co-occurring alcohol or substance use issues in trials of psychological interventions for psychosis and aim to measure alcohol or substance use, both at baseline and at follow-up, to enable the efficacy of psychological interventions for psychosis for individuals with co-occurring A/SUD to be determined. Resultingly, clinical guidelines for the management of psychosis may then evidentially account for individuals with co-occurring A/SUD, supporting the delivery of suitable and effective treatment for these patients and therefore ultimately improving outcomes.

## Supplementary Material

sbae073_suppl_Supplementary_Material

## References

[1] Brunette MF , Mueser KT, Babbin S, et al Demographic and clinical correlates of substance use disorders in first episode psychosis. Schizophr Res. 2018;194:4–12. 28697856 10.1016/j.schres.2017.06.039

[2] Hunt GE , Large MM, Cleary M, Lai HMX, Saunders JB. Prevalence of comorbid substance use in schizophrenia spectrum disorders in community and clinical settings, 1990–2017: systematic review and meta-analysis. Drug Alcohol Depend. 2018;191:234–258. 30153606 10.1016/j.drugalcdep.2018.07.011

[3] Sara GE , Burgess PM, Malhi GS, Whiteford HA, Hall WC. Stimulant and other substance use disorders in schizophrenia: prevalence, correlates and impacts in a population sample. Aust N Z J Psychiatry. 2014;48:1036–1047. 24819935 10.1177/0004867414533838

[4] Dixon L. Dual diagnosis of substance abuse in schizophrenia: prevalence and impact on outcomes. Schizophr Res. 1999;35:S93–S100. 10190230 10.1016/s0920-9964(98)00161-3

[5] Heiberg IH , Jacobsen BK, Nesvåg R, et al Total and cause-specific standardized mortality ratios in patients with schizophrenia and/or substance use disorder. PLoS One. 2018;13:e0202028. 30138449 10.1371/journal.pone.0202028 PMC6107156

[6] Lähteenvuo M , Batalla A, Luykx JJ, et al Morbidity and mortality in schizophrenia with comorbid substance use disorders. Acta Psychiatr Scand. 2021;144:42–49. 33650123 10.1111/acps.13291 PMC8359349

[7] National Institute for Health and Care Excellence. Coexisting Severe Mental Illness (Psychosis) and Substance Misuse: Assessment and Management in Healthcare Settings. London: National Institute for Health and Care Excellence; 2011. 31851442

[8] National Institute for Health and Care Excellence. Psychosis and Schizophrenia in Adults: Prevention and Management. London: National Institute for Health and Care Excellence; 2014. 32207892

[9] McGlanaghy E , Turner D, Davis GA, et al A network meta-analysis of psychological interventions for schizophrenia and psychosis: impact on symptoms. Schizophr Res. 2021;228:447–459. 33578368 10.1016/j.schres.2020.12.036

[10] Tinetti ME , Basu J. Research on multiple chronic conditions: where we are and where we need to go. Med Care. 2014;52:S3–S6. 10.1097/MLR.0000000000000093 24561755

[11] Stoll CR , Izadi S, Fowler S, et al Multimorbidity in randomized controlled trials of behavioral interventions: a systematic review. Health Psychol. 2019;38:831–857. 31045382 10.1037/hea0000726 PMC6983953

[12] Leeman RF , Hefner K, Frohe T, et al Exclusion of participants based on substance use status: findings from randomized controlled trials of treatments for PTSD. Behav Res Ther. 2017;89:33–40. 27846419 10.1016/j.brat.2016.10.006

[13] Tinetti ME , Fried TR, Boyd CM. Designing health care for the most common chronic condition—multimorbidity. JAMA. 2012;307:2493–2494. 22797447 10.1001/jama.2012.5265 PMC4083627

[14] Ziedonis DM , Bizamcer AN, Steinberg ML, Wyatt SA, Smelson S, Vaiana AD. Co-occurring addiction and psychotic disorders. In: Reis R, Fiellin D, Miller S, et al., eds. Principles of Addiction Medicine. 4th ed. New York, NY: American Society of Addiction Medicine; 2009:1193–1209.

[15] Smith SM , Soubhi H, Fortin M, Hudon C, O’Dowd T. Managing patients with multimorbidity: systematic review of interventions in primary care and community settings. BMJ. 2012;345:e5205. 22945950 10.1136/bmj.e5205 PMC3432635

[16] Karapareddy V. A review of integrated care for concurrent disorders: cost effectiveness and clinical outcomes. J Dual Diagn. 2019;15:56–66. 30806190 10.1080/15504263.2018.1518553

[17] Martinez CP. Co-occurring psychiatric disorders. In: Marienfeld C, ed. Absolute Addiction Psychiatry Review. San Diego, CA: Springer; 2020:335–348.

[18] Roberts NP , Lotzin A, Schäfer I. A systematic review and meta-analysis of psychological interventions for comorbid post-traumatic stress disorder and substance use disorder. Eur J Psychotraumatol. 2022;13:1–23. 10.1080/20008198.2022.2041831 PMC9090345 35558682

[19] Page MJ , McKenzie JE, Bossuyt PM, et al The PRISMA 2020 statement: an updated guideline for reporting systematic reviews. Int J Surg. 2021;10:105906. 10.1016/j.ijsu.2021.105906 33789826

[20] Hariton E , Locascio JJ. Randomised controlled trials—the gold standard for effectiveness research. BJOG. 2018;125:1716–1721. 29916205 10.1111/1471-0528.15199 PMC6235704

[21] Richardson WS , Wilson MC, Nishikawa J, et al The well-built clinical question: a key to evidence-based decisions. ACP J Club. 1995;123:12–23. 10.7326/ACPJC-1995-123-3-A12 7582737

[22] Buckley PF , Miller BJ, Lehrer DS, Hayward RS. Psychiatric comorbidities and schizophrenia. Schizophr Bull. 2009;35:383–402. 19011234 10.1093/schbul/sbn135 PMC2659306

[23] Tsai J , Rosenheck RA. Psychiatric comorbidity among adults with schizophrenia: a latent class analysis. Psychiatry Res. 2013;210:16–20. 23726869 10.1016/j.psychres.2013.05.013 PMC3800495

[24] Bramley G , Fitzpatrick S, Edwards J, et al. Hard Edges: Mapping Severe and Multiple Disadvantage in England. London: Lankelley Chase Foundation; 2015.

[25] Fazel S , Bains P, Doll H. Substance abuse and dependence in prisoners: a systematic review. Addiction. 2006;101:181–191. 16445547 10.1111/j.1360-0443.2006.01316.x

[26] Kay SR , Fiszbein A, Opler LA. The positive and negative syndrome scale (PANSS) for schizophrenia. Schizophr Bull. 1987;13:261–276. 3616518 10.1093/schbul/13.2.261

[27] Overall JE , Gordham DR. The brief psychiatric rating scale. Psychol Rep. 1962;10:790–812.

[28] Guy W. Clinical Global Impression, ECDEU Assessment Manual for Psychopharmacology, Revised. Rockville, MD: National Institute of Mental Health; 1976.

[29] Li T , Higgins JPT, Deeks JJ. Chapter 5: collecting data. In: Li T, Page MJ, Welch VA, eds. Cochrane Handbook for Systematic Reviews of Interventions. Cochrane; 2023. www.training.cochrane.org/handbook

[30] Sterne JAC , Savović J, Page MJ, et al RoB2: a revised tool for assessing risk of bias in randomised trials. BMJ. 2019;366:14898. 10.1136/bmj.l4898 31462531

[31] Campbell M , McKenzie JE, Sowden A, et al Synthesis without meta-analysis (SWiM) in systematic reviews: reporting guideline. BMJ. 2020;368:l6890. 31948937 10.1136/bmj.l6890 PMC7190266

[32] McKenzie JE , Brennan S. Chapter 12: synthesizing and presenting findings using other methods. In: Higgins JPT, Thomas J, Chandler J, et al., eds. Cochrane Handbook for Systematic Reviews of Interventions. Cochrane; 2023. www.training.cochrane.org/handbook

[33] Boon MH , Thomson H. The effect direction plot revisited: application of the 2019 Cochrane Handbook guidance on alternative synthesis methods. Res Synth Methods. 2021;12:29–33. 32979023 10.1002/jrsm.1458 PMC7821279

[34] Thomson HJ , Thomas S. The effect direction plot: visual display of non-standardised effects across multiple outcome domains. Res Synth Methods. 2013;4:95–101. 23795209 10.1002/jrsm.1060 PMC3688329

[35] Dalum HS , Waldemar AK, Korsbek L, et al Illness management and recovery: clinical outcomes of a randomized clinical trial in community mental health centers. PLoS One. 2018;13:e0194027–e0194016. 29621284 10.1371/journal.pone.0194027 PMC5886399

[36] Morrison AP , Pyle M, Gumley A, et al Cognitive-behavioural therapy for clozapine-resistant schizophrenia: the FOCUS RCT. Health Technol Assess. 2019;23:1–144. 10.3310/hta23070 PMC6409493 30806619

[37] Gleeson JF , Cotton SM, Alvarez-Jimenez M, et al. A randomized controlled trial of relapse prevention therapy for first-episode psychosis patients: outcome at 30-month follow-up. *Schizophr Bull*. 2013;39:436–448. 22130905 10.1093/schbul/sbr165 PMC3576162

[38] Aho-Mustonen K , Tiihonen J, Repo-Tiihonen E, Ryynänen OP, Miettinen R, Räty H. Group psychoeducation for long-term offender patients with schizophrenia: an exploratory randomised controlled trial. Crim Behav Ment Health. 2011;21:163–176. 20859932 10.1002/cbm.788

[39] Favrod J , Rexhaj S, Bardy S, et al. Sustained antipsychotic effect of metacognitive training in psychosis: a randomized-controlled study. *Euro Psychiatry*. 2014;29:275–281. 10.1016/j.eurpsy.2013.08.003 24176646

[40] Kuokkanen P , Lappalainen R, Repo-Tiihonen R, Tiihonen J. Metacognitive group training and forensic and dangerous non-forensic patients with schizophrenia: A randomised controlled feasibility trial. *Crim Behav Ment Health*. 2014;24:345–357. 24619628 10.1002/cbm.1905

[41] Moritz S , Kerstan A, Veckenstedt R, et al. Further evidence for the efficacy of a metacognitive group training in schizophrenia. *Behav Res Ther*. 2011;49:151–147. 21276962 10.1016/j.brat.2010.11.010

[42] Khazaal Y , Chatton A, Dieben K, et al Reducing delusional conviction through a cognitive-based group training game: a multicentre randomized controlled trial. Front Psychiatry. 2015;6:66–77. 25972817 10.3389/fpsyt.2015.00066 PMC4412136

[43] Secher RG , Hjorthøj CR, Austin SF, et al Ten-year follow-up of the OPUS specialized early intervention trial for patients with a first episode of psychosis. Schizophr Bull. 2015;41:617–626. 25381449 10.1093/schbul/sbu155 PMC4393691

[44] Du Vaure CB , Dechartres A, Battin C, Ravaud P, Boutron I. Exclusion of patients with concomitant chronic conditions in ongoing randomised controlled trials targeting 10 common chronic conditions and registered at ClinicalTrials. gov: a systematic review of registration details. BMJ Open. 2016;6:e012265. 10.1136/bmjopen-2016-012265 PMC5051474 27678540

[45] Jadad AR , To MJ, Emara M, Jones J. Consideration of multiple chronic diseases in randomized controlled trials. JAMA. 2011;306:2670–2672. 10.1001/jama.2011.1886 22203536

[46] Van Spall HG , Toren A, Kiss A, Fowler RA. Eligibility criteria of randomized controlled trials published in high-impact general medical journals: a systematic sampling review. JAMA. 2007;297:1233–1240. 17374817 10.1001/jama.297.11.1233

[47] de Wet A , Swartz L, Chiliza B. Hearing their voices: the lived experience of recovery from first-episode psychosis in schizophrenia in South Africa. Int J Soc Psychiatry. 2015;61:27–32. 24874119 10.1177/0020764014535753

[48] Pitt L , Kilbride M, Nothard S, Welford M, Morrison AP. Researching recovery from psychosis: a user-led project. Psychiatr Bull. 2007;31:55–60.

[49] Wood L , Alsawy S. Recovery in psychosis from a service user perspective: a systematic review and thematic synthesis of current qualitative evidence. Community Ment Health J. 2018;54:793–804. 29188393 10.1007/s10597-017-0185-9

