# Supplementary material for: Substance Use Within Trials of Psychological Interventions for Psychosis: Sample Inclusion, Secondary Measures, and Intervention Effectiveness
Source: Schizophr Bull. 2024 May 23;50(6):1489–98. doi: 10.1093/schbul/sbae073 (PMC11548936; doi:10.1093/schbul/sbae073)
Supplement: sbae073_suppl_Supplementary_Material [file sbae073_suppl_supplementary_material.docx]

**Supplementary Materials**

**Psychological intervention type**

A classification of psychological intervention type for all psychological interventions included in the full review.

| Intervention Type | Code | Description |
| --- | --- | --- |
| Cognitive behavioral therapy | CBT | CBT is a psychotherapeutic intervention focused upon the association between thoughts, feelings, and somatic sensation. Typically, CBT includes some aspects of psychoeducation, challenging negative cognition, and relaxation techniques, as well as behavioral activities. |
| Metacognitive therapy | MCT | MCT is a form of CBT that focuses specifically on metacognitions, and how to modify maladaptive metacognitive beliefs. As with traditional CBT, MCT likely includes some aspects of psychoeducation, challenging negative metacognition, and may also include relaxation techniques. |
| OPUS early intervention service | OPUS | OPUS treatment is a specific, psychological, early intervention service, that incorporates modified assertive community treatment, SST, and PE. |
| Psychoeducation and coping | PE | PE is the sharing of information or knowledge to help patients better understand their disorder, symptoms, or experiences. Focus may also be placed on coping specifically. |
| Social skills training* | SST | SST is a based on social learning theory, and focuses upon vicarious and reinforced learning of social behavior and social functioning, to reduce social distress and manage daily life. |

* = not investigated within any of the trials included within the full review directly, but described as it is a component of another intervention which was explicitly investigated within a trial(s) included within the full review.

**Risk of bias assessment**

Risk of bias assessment for all trials included within the full review.

| Citation | Randomisation | Deviation from intended intervention | Missing data | Outcome measurement | Result reporting | Overall |
| --- | --- | --- | --- | --- | --- | --- |
| Aho-Mustonen (2011) |  |  |  |  |  |  |
| Favrod (2014) |  |  |  |  |  |  |
| Gleeson (2013) |  |  |  |  |  |  |
| Khazaal (2015) |  |  |  |  |  |  |
| Kuokkanen (2014) |  |  |  |  |  |  |
| Moritz (2011) |  |  |  |  |  |  |
| Secher (2015) |  |  |  |  |  |  |

Red = high risk of bias, amber = some concerns, green = low risk of bias.

**Reference list of included studies**

Abaoğlu H, Mutlu E, Ak S, Aki E, Yağcioğlu AAYSE. The effect of life skills training on functioning in schizophrenia: A randomised controlled trial. *Schizophr Bull* 2020;46:220-230.

Acuña V, Otto A, Cavieres A, Villalobos H. Efficacy of metacognitive training in a Chilean sample of people with schizophrenia. *Revista Colombiana de psiquiatria (English ed.)* 2022;51:301-308.

Aho‐Mustonen K, Tiihonen J, Repo‐Tiihonen E, Ryynänen OP, Miettinen R, Räty H. Group psychoeducation for long‐term offender patients with schizophrenia: An exploratory randomised controlled trial. *Crim Behav Ment Health* 2011;21:163-176.

Aloi M, de Filippis R, Lavalle FG, et al. Effectiveness of integrated psychological therapy on clinical, neuropsychological, emotional and functional outcome in schizophrenia: a RCT study. *J Ment Health* 2018;29:524-513.

Awan NR, Jehangir SF, Irfan M, Naeem F, Farooq S. Explanatory model of illness of the patients with schizophrenia and the role of educational intervention. *Schizophr Res* 2017;190:68-73.

Bartels SJ, Pratt SI, Mueser KT, et al. Long-term outcomes of a randomized trial of integrated skills training and preventive healthcare for older adults with serious mental illness. *Am J Geriatr Psychiatry* 2014;22:1251-1261.

Beauchamp MC, Lecomte T, Lecomte C, Leclerc C, Corbière M. Do personality traits matter when choosing a group therapy for early psychosis?. *Psychol Psychother* 2013;86:19-32.

Birchwood M, Michail M, Meaden A, et al. Cognitive behaviour therapy to prevent harmful compliance with command hallucinations (COMMAND): a randomised controlled trial. *Lancet Psychiatry* 2014;1:23-33.

Bossert M, Westermann C, Schilling TM, Weisbrod M, Roesch-Ely D, Aschenbrenner S. Computer-assisted cognitive remediation in schizophrenia: efficacy of an individualized vs. Generic exercise plan. *Front Psychiatry* 2020;11:555052.

Braehler C, Gumley A, Harper J, Wallace S, Norrie J, Gilbert P. Exploring change processes in compassion focused therapy in psychosis: Results of a feasibility randomized controlled trial. *Br J Clin Psychol* 2013;52:199-214.

Briki M, Monnin J, Haffen E, et al. Metacognitive training for schizophrenia: a multicentre randomised controlled trial. *Schizophr Res* 2014;157:99-106.

Byrne LK, Peng D, McCabe M, et al. Does practice make perfect? Results from a Chinese feasibility study of cognitive remediation in schizophrenia. *Neuropsychol Rehabil* 2013;23:580-596.

Cai J, Zhu Y, Zhang W, Wang Y, Zhang C. Comprehensive family therapy: an effective approach for cognitive rehabilitation in schizophrenia. *Neuropsychiatr Dis Treat* 2015;11:1247-1253.

Cassetta BD, Tomfohr-Madsen LM, Goghari VM. A randomized controlled trial of working memory and processing speed training in schizophrenia. *Psychol Med*  2019;49:2009-2019.

Cella M, Reeder C, Wykes T. It is all in the factors: effects of cognitive remediation on symptom dimensions. *Schizophr Res* 2014;156:60-62.

Cella M, Tomlin P, Robotham D, et al. Virtual reality therapy for the negative symptoms of schizophrenia (V-NeST): a pilot randomised feasibility trial. *Schizophr Res* 2022;248:50-57.

Chadwick P, Strauss C, Jones AM, et al. Group mindfulness-based intervention for distressing voices: a pragmatic randomised controlled trial. *Schizophr Res* 2016;175:168-173.

Chien WT, Lee IY. The mindfulness-based psychoeducation program for Chinese patients with schizophrenia. *Psychiatr Serv* 2013;64:376-379.

Chien WT, Thompson DR. An RCT with three-year follow-up of peer support groups for Chinese families of persons with schizophrenia. *Psychiatr Serv* 2013;64:997-1005.

Chien WT, Thompson DR. Effects of a mindfulness-based psychoeducation programme for Chinese patients with schizophrenia: 2-year follow-up. *Br J Psychiatry* 2014;205:52-59.

Chien WT, Bressington D. A randomized controlled clinical trial of a nurse-led structured psychosocial intervention program for people with first-onset mental illness in psychiatric outpatient clinics. *Psychiatry Res* 2015;229:277-286.

Chien WT, Bressington D, Yip A, Karatzias T. An international multi-site, randomized controlled trial of a mindfulness-based psychoeducation group programme for people with schizophrenia. *Psychol Med* 2017;47:2081-2096.

Chien WT, Cheng HY, McMaster TW, Yip AL, Wong JC. Effectiveness of a mindfulness-based psychoeducation group programme for early-stage schizophrenia: An 18-month randomised controlled trial. *Schizophr Res* 2019;212:140-149.

Chien WT, Ho LK, Gray R, Bressington D. A randomized controlled trial of a peer-facilitated self-management program for people with recent-onset psychosis. *Schizophr Res* 2022;250:22-30.

Chen Q, Sang Y, Ren L, et al. Metacognitive training: a useful complement to community-based rehabilitation for schizophrenia patients in China. *BMC Psychiatry* 2021;21:1-10.

Choi KH, Kang J, Kim SM, et al. Cognitive remediation in middle-aged or older inpatients with chronic schizophrenia: a randomized controlled trial in Korea. *Front Psychol* 2018;8:2364.

Craig TK, Rus-Calafell M, Ward T, et al. AVATAR therapy for auditory verbal hallucinations in people with psychosis: a single-blind, randomised controlled trial. *Lancet Psychiatry* 2018;5:31-40.

Dalum HS, Waldemar AK, Korsbek L, et al. Illness management and recovery: clinical outcomes of a randomized clinical trial in community mental health centers. *PloS ONE* 2018;13:e0194027.

d'Amato T, Bation R, Cochet A, et al. A randomized, controlled trial of computer-assisted cognitive remediation for schizophrenia. *Schizophr Res* 2011;125:284-290.

de Pinho LM, da Cruz Sequeira CA, Miguel F, et al. Assessing the efficacy and feasibility of providing metacognitive training for patients with schizophrenia by mental health nurses: A randomized controlled trial. *J Adv Nurs* 2020;00:1-4.

Depp CA, Perivoliotis D, Holden J, Dorr J, Granholm EL. Single-session mobile-augmented intervention in serious mental illness: a three-arm randomized controlled trial. *Schizophr Bull* 2019;45:752-762.

Dunn G, Fowler D, Rollinson R, et al. Effective elements of cognitive behaviour therapy for psychosis: results of a novel type of subgroup analysis based on principal stratification. *Psychol Med* 2012:42:1057-1068.

Du Sert OP, Potvin S, Lipp O, et al. Virtual reality therapy for refractory auditory verbal hallucinations in schizophrenia: a pilot clinical trial. *Schizophr Res* 2018;197:176-181.

El Ashry AM, Abd El Dayem SM, Ramadan FH. Effect of applying “acceptance and commitment therapy” on auditory hallucinations among patients with schizophrenia. *Arch Psychiatr Nurs* 2021;35:141-152.

Fan F, Zou Y, Tan Y, Hong LE, Tan S. Computerized cognitive remediation therapy effects on resting state brain activity and cognition in schizophrenia. *Sci Rep* 2017;7:4758-4764.

Färdig R, Lewander T, Melin L, Folke F, Fredriksson A. A randomized controlled trial of the illness management and recovery program for persons with schizophrenia. *Psychiatr Serv* 2011;62:606-612.

Favrod J, Nguyen A, Chaix J, et al. Improving pleasure and motivation in schizophrenia: a randomized controlled clinical trial. *Psychother Psychosom* 2019;88:84-95.

Favrod J, Rexhaj S, Bardy S, et al. Sustained antipsychotic effect of metacognitive training in psychosis: A randomized-controlled study. *Eur Psychiatry* 2014;29:275-281.

Fekete Z, Vass E, Balajthy R, et al. Efficacy of metacognitive training on symptom severity, neurocognition and social cognition in patients with schizophrenia: A single‐blind randomized controlled trial. *Scand J Psychol* 2022;63:321-333.

Fiszdon JM, Choi KH, Bell MD, Choi J, Silverstein SM. Cognitive remediation for individuals with psychosis: efficacy and mechanisms of treatment effects. *Psychol Med* 2016;46:3275-3289.

Freeman D, Pugh K, Dunn G, et al. An early Phase II randomised controlled trial testing the effect on persecutory delusions of using CBT to reduce negative cognitions about the self: the potential benefits of enhancing self confidence. *Schizophr Res* 2014;160:186-192.

Freeman D, Dunn G, Startup H, et al. Effects of cognitive behaviour therapy for worry on persecutory delusions in patients with psychosis (WIT): a parallel, single-blind, randomised controlled trial with a mediation analysis. *Lancet Psychiatry* 2015;2:305-313.

Garety P, Ward T, Emsley R, et al. Effects of SlowMo, a blended digital therapy targeting reasoning, on paranoia among people with psychosis: A randomized clinical trial. *JAMA Psychiatry* 2021;78:714-725.

Gharaeipour M, Scott BJ. Effects of cognitive remediation on neurocognitive functions and psychiatric symptoms in schizophrenia inpatients. *Schizophr Res* 2012;142:165-170.

Gleeson JF, Cotton SM, Alvarez-Jimenez M, et al. A randomized controlled trial of relapse prevention therapy for first-episode psychosis patients: outcome at 30-month follow-up. *Schizophr Bull* 2013;39:436-448.

Goldsmith LP, Lewis SW, Dunn G, Bentall RP. Psychological treatments for early psychosis can be beneficial or harmful, depending on the therapeutic alliance: an instrumental variable analysis. *Psychol Med* 2015;45:2365-2373.

Gottlieb JD, Gidugu V, Maru M, et al. Randomized controlled trial of an internet cognitive behavioral skills-based program for auditory hallucinations in persons with psychosis. *Psychiatr Rehabil J* 2017;40:283-293.

Granholm E, Holden J, Link PC, McQuaid JR, Jeste DV. Randomized controlled trial of cognitive behavioral social skills training for older consumers with schizophrenia: defeatist performance attitudes and functional outcome. *Am J Geriatr Psychiatr* 2013;21:251-262.

Granholm E, Twamley EW, Mahmood Z, et al. Integrated cognitive-behavioral social skills training and compensatory cognitive training for negative symptoms of psychosis: effects in a pilot randomized controlled trial. *Schizophr Bull* 2022;48:359-370.

Grant PM, Bredemeier K, Beck AT. Six-month follow-up of recovery-oriented cognitive therapy for low-functioning individuals with schizophrenia. *Psychiatr Serv* 2017;68:997-1002.

Habib N, Dawood S, Kingdon D, Naeem F. Preliminary evaluation of culturally adapted CBT for psychosis (CA-CBTp): findings from developing culturally-sensitive CBT project (DCCP). *Behav Cogn Psychother* 2015;43:200-208.

Hasan AA, Callaghan P, Lymn JS. Evaluation of the impact of a psycho-educational intervention for people diagnosed with schizophrenia and their primary caregivers in Jordan: a randomized controlled trial. *BMC Psychiatry* 2015;15:1-10.

Hatami S, Mirsepassi Z, Sedighnia A, Tehranidoost M, Masoomi M, Sharifi V. A short course computer-assisted cognitive remediation in patients with schizophrenia spectrum disorders: A randomized clinical trial. *Basic Clin Neurosci* 2021;12:551-563.

Haugen I, Stubberud J, Haug E, et al. A randomized controlled trial of Goal Management Training for executive functioning in schizophrenia spectrum disorders or psychosis risk syndromes. *BMC Psychiatry* 2022;22:575-590.

Hegde S, Rao SL, Raguram A, Gangadhar BN. Addition of home-based cognitive retraining to treatment as usual in first episode schizophrenia patients: A randomized controlled study. *Indian J Psychiatry* 2012;54:15.

Husain MO, Chaudhry IB, Mehmood N, et al. Pilot randomised controlled trial of culturally adapted cognitive behavior therapy for psychosis (CaCBTp) in Pakistan. *BMC Health Serv Res* 2017;17:1-8.

Ishikawa R, Ishigaki T, Shimada T, et al. The efficacy of extended metacognitive training for psychosis: A randomized controlled trial. *Schizophr Res* 2020;215:399-407.

Iwata K, Matsuda Y, Sato S, et al. Efficacy of cognitive rehabilitation using computer software with individuals living with schizophrenia: A randomized controlled trial in Japan. *Psychiatr Rehabil J* 2017;40:4-11.

Kanie A, Kikuchi A, Haga D, et al. The feasibility and efficacy of social cognition and interaction training for outpatients with schizophrenia in Japan: a multicenter randomized clinical trial. *Front Psychiatry* 2019;10:589-597.

Kariofillis D, Sartory G, Kärgel C, Müller BW. The effect of cognitive training on evoked potentials in schizophrenia. *Schizophr Res Cogn* 2014;1:180-186.

Katsumi A, Hoshino H, Fujimoto S, et al. Effects of cognitive remediation on cognitive and social functions in individuals with schizophrenia. *Neuropsychol Rehabil* 2017;29:1475-1487.

Khalil AH, ELNahas G, Ramy H, Abdel Aziz K, Elkholy H, El-Ghamry R. Impact of a culturally adapted behavioural family psychoeducational programme in patients with schizophrenia in Egypt. *Int J Psychiatry Clin Pract* 2019;23:62-71.

Khazaal Y, Chatton A, Dieben K et al. Reducing delusional conviction through a cognitive-based group training game: a multicentre randomized controlled trial. *Front Psychiatry* 2015;6:66-77.

Knott V, Wright N, Shah D, et al. Change in the neural response to auditory deviance following cognitive therapy for hallucinations in patients with schizophrenia. *Front Psychiatry* 2020;11:555-567.

Kråkvik B, Gråwe RW, Hagen R, Stiles TC. Cognitive behaviour therapy for psychotic symptoms: a randomized controlled effectiveness trial. *Behav Cogn Psychother* 2013;41:511-524.

Kuokkanen R, Lappalainen R, Repo‐Tiihonen E, Tiihonen J. Metacognitive group training for forensic and dangerous non‐forensic patients with schizophrenia: A randomised controlled feasibility trial. *Crim Behav Ment Health* 2014;24:345-357.

Lam AHY, Leung SF, Lin JJ, Chien WT. The effectiveness of a mindfulness-based psychoeducation programme for emotional regulation in individuals with schizophrenia spectrum disorders: a pilot randomised controlled trial. *Neuropsychiatr Dis Treat* 2020;16:729-747.

Lee RS, Redoblado-Hodge MA, Naismith SL, et al. Cognitive remediation improves memory and psychosocial functioning in first-episode psychiatric out-patients*. Psychol Med* 2013;43:1161-1173.

Lee KH. A randomized controlled trial of mindfulness in patients with schizophrenia. *Psychiatry Res* 2019;275:137-142.

Lee WK. Effectiveness of computerized cognitive rehabilitation training on symptomatological, neuropsychological and work function in patients with schizophrenia. *Asia‐Pac Psychiatry* 2013;5:90-100.

Leff J, Williams G, Huckvale MA, Arbuthnot M, Leff AP. Computer-assisted therapy for medication-resistant auditory hallucinations: proof-of-concept study. *Br J Psychiatry* 2013;202:428-433.

Lewandowski KE, Eack SM, Hogarty SS, Greenwald DP, Keshavan MS. Is cognitive enhancement therapy equally effective for patients with schizophrenia and schizoaffective disorder?. *Schizophr Res* 2011;125:291-294.

Li ZJ, Guo ZH, Wang N, et al. Cognitive–behavioural therapy for patients with schizophrenia: a multicentre randomized controlled trial in Beijing, China. *Psychol Med* 2015;45:1893-1905.

Lincoln TM, Ziegler M, Mehl S, et al. Moving from efficacy to effectiveness in cognitive behavioral therapy for psychosis: a randomized clinical practice trial. *J Consult Clin Psychol* 2012;80:674-686.

Liu Y, Yang X, Gillespie A, et al. Targeting relapse prevention and positive symptom in first-episode schizophrenia using brief cognitive behavioral therapy: A pilot randomized controlled study. *Psychiatry Res* 2019;272:275-283.

Longden E, Corstens D, Bowe S, et al. A psychological intervention for engaging dialogically with auditory hallucinations (Talking With Voices): A single-site, randomised controlled feasibility trial. *Schizophr Res* 2022;250:172-179.

Luo X, Law SF, Wang X, et al. Effectiveness of an assertive community treatment program for people with severe schizophrenia in mainland China–a 12-month randomized controlled trial. *Psychol Med* 2019;49:969-979.

Matsuda Y, Morimoto T, Furukawa S, et al. Feasibility and effectiveness of a cognitive remediation programme with original computerised cognitive training and group intervention for schizophrenia: a multicentre randomised trial. *Neuropsychol Rehabil* 2018;28:387-397.

Matsuoka K, Morimoto T, Matsuda Y, et al. Computer-assisted cognitive remediation therapy for patients with schizophrenia induces microstructural changes in cerebellar regions involved in cognitive functions. *Psychiatry Res Neuroimaging* 2019;292:41-46.

Mendella PD, Burton CZ, Tasca GA, Roy P, Louis LS, Twamley EW. Compensatory cognitive training for people with first-episode schizophrenia: results from a pilot randomized controlled trial. *Schizophr Res* 2015;162:108-111.

Morimoto T, Matsuda Y, Matsuoka K, et al. Computer-assisted cognitive remediation therapy increases hippocampal volume in patients with schizophrenia: a randomized controlled trial. *BMC Psychiatry* 2018;18:1-8.

Moritz S, Kerstan A, Veckenstedt R, et al. Further evidence for the efficacy of a metacognitive group training in schizophrenia. *Behav Res Ther* 2011;49:151-157.

Morrison AP, Turkington D, Pyle M, et al. Cognitive therapy for people with schizophrenia spectrum disorders not taking antipsychotic drugs: a single-blind randomised controlled trial. *Lancet* 2014;383:1395-1403.

Morrison AP, Pyle M, Gumley A, et al. Cognitive-behavioural therapy for clozapine-resistant schizophrenia: The FOCUS RCT. *Health Technol Assess* 2019;23.

Mueller DR, Schmidt SJ, Roder V. One-year randomized controlled trial and follow-up of integrated neurocognitive therapy for schizophrenia outpatients. *Schizophr Bull* 2015;41:604-616.

Mueller DR, Khalesi Z, Benzing V, Castiglione CI, Roder V. Does Integrated Neurocognitive Therapy (INT) reduce severe negative symptoms in schizophrenia outpatients?. *Schizophr Res* 2017;188:92-97.

Mueller DR, Khalesi Z, Roder V. Can cognitive remediation in groups prevent relapses?: results of a 1-year follow-up randomized controlled trial. *J Nerv Ment* 2020;208:362-370.

Muhić M, Janković S, Sikira H, et al. Multifamily groups for patients with schizophrenia: an exploratory randomised controlled trial in Bosnia and Herzegovina. *Soc Psychiatry Psychiatr Epidemiol* 2022;57:1357-1364.

Myin-Germeys I, van Aubel E, Vaessen T, et al. Efficacy of acceptance and commitment therapy in daily life in early psychosis: results from the multi-center INTERACT randomized controlled trial. *Psychother Psychosom* 2022;91:411-423.

Naeem F, Saeed S, Irfan M, et al. Brief culturally adapted CBT for psychosis (CaCBTp): a randomized controlled trial from a low income country. *Schizophr Res* 2015;164:143-148.

Naeem F, Johal R, McKenna C, et al. Cognitive Behavior Therapy for psychosis based Guided Self-help (CBTp-GSH) delivered by frontline mental health professionals: Results of a feasibility study. *Schizophr Res* 2016;173:69-74.

Omiya H, Yamashita K, Miyata T, et al. Pilot study of the effects of cognitive remediation therapy using the frontal/executive program for treating chronic schizophrenia. *Open Psychol J* 2016;9:121-128.

O’Reilly K, Donohoe G, O’Sullivan D, et al. A randomized controlled trial of cognitive remediation for a national cohort of forensic patients with schizophrenia or schizoaffective disorder. *BMC Psychiatry* 2019;19:1-12.

Palma C, Farriols N, Frías A, et al. Randomized controlled trial of cognitive-motivational therapy program (PIPE) for the initial phase of schizophrenia: Maintenance of efficacy at 5-year follow up. *Psychiatry Res* 2019; 273:586-594.

Palumbo D, Caporusso E, Piegari G, et al. Social cognition individualized activities lab for social cognition training and narrative enhancement in patients with schizophrenia: A randomized controlled study to assess efficacy and generalization to real-life functioning. *Front Psychiatry* 2022;13:833550.

Pos K, Franke N, Smit F, et al. Cognitive behavioral therapy for social activation in recent-onset psychosis: Randomized controlled trial. *J Consult Clin Psychol* 2019;87:151-160.

Rajji TK, Mamo DC, Holden J, Granholm E, Mulsant BH. Cognitive-Behavioral Social Skills Training for patients with late-life schizophrenia and the moderating effect of executive dysfunction. *Schizophr Res* 2022;239:160-167.

Rakitzi S, Georgila P, Efthimiou K, Mueller DR. Efficacy and feasibility of the Integrated Psychological Therapy for outpatients with schizophrenia in Greece: Final results of a RCT. *Psychiatry Res* 2016;242:137-143.

Rathod S, Phiri P, Harris S, et al. Cognitive behaviour therapy for psychosis can be adapted for minority ethnic groups: a randomised controlled trial. *Schizophr Res* 2013;143:319-326.

Reeder C, Huddy V, Cella M, et al. A new generation computerised metacognitive cognitive remediation programme for schizophrenia (CIRCuiTS): a randomised controlled trial. *Psychol Med* 2017;47:2720-2730.

Roberts DL, Combs DR, Willoughby M, et al. A randomized, controlled trial of Social Cognition and Interaction Training (SCIT) for outpatients with schizophrenia spectrum disorders. *Br J Clin Psychol* 2014;53:281-298.

Ruggeri M, Bonetto C, Lasalvia A, et al. Feasibility and effectiveness of a multi-element psychosocial intervention for first-episode psychosis: results from the cluster-randomized controlled GET UP PIANO trial in a catchment area of 10 million inhabitants. *Schizophr Bull* 2015;41:1192-1203.

Rus-Calafell M, Gutiérrez-Maldonado J, Ortega-Bravo M, Ribas-Sabaté J, Caqueo-Urízar A. A brief cognitive–behavioural social skills training for stabilised outpatients with schizophrenia: A preliminary study. *Schizophr Res* 2013;143:327-336.

Sachs G, Winklbaur B, Jagsch R, et al. Training of affect recognition (TAR) in schizophrenia—impact on functional outcome. *Schizophr Res* 2012;138:262-267.

Schaub A, Mueser KT, von Werder T, Engel R, Möller HJ, Falkai P. A randomized controlled trial of group coping-oriented therapy vs supportive therapy in schizophrenia: results of a 2-year follow-up. *Schizophr Bull* 2016;42:71-80.

Schlosser DA, Campellone TR, Truong B, et al. Efficacy of PRIME, a mobile app intervention designed to improve motivation in young people with schizophrenia. *Schizophr Bull* 2018;44:1010-1020.

Schnackenberg J, Fleming M, Martin CR. A randomised controlled pilot study of Experience Focused Counselling with voice hearers. *Psychosis* 2017;9:12-24.

Schrank B, Brownell T, Jakaite Z, et al. Evaluation of a positive psychotherapy group intervention for people with psychosis: pilot randomised controlled trial. *Epidemiol Psychiatr Sci* 2016;25:235-246.

Secher RG, Hjorthøj CR, Austin SF, et al. Ten-year follow-up of the OPUS specialized early intervention trial for patients with a first episode of psychosis. *Schizophr Bull* 2015;41:617-626.

Sevi MO, Sutcu TS, Yesilyurt S, Eroguli TS, Gunes B. Comparison of the effectiveness of two cognitive-behavioral group therapy programs for schizophrenia: Results of a short-term randomized control trial. *Community Ment Health J* 2019;56:222-228.

Singh J, Singh S, Chavan BS, et al. Efficacy of Cognitive Training Program Given to Patients with Schizophrenia Using Computer Tablets: A Preliminary Study. *Int J Cogn Therap* 2023;16:40-57.

Siu AM, Ng RS, Poon MY, Chong CS, Siu CM, Lau SP. Evaluation of a computer-assisted cognitive remediation program for young people with psychosis: a pilot study. *Schizophr Res Cogn* 2021;23:100188.

So SHW, Chan AP, Chong CSY, et al. Metacognitive training for delusions (MCTd): effectiveness on data-gathering and belief flexibility in a Chinese sample. *Front Psychol* 2015;6:730-745.

Stiekema AP, van Dam MT, Bruggeman R, et al. Facilitating recovery of daily functioning in people with a severe mental illness who need longer-term intensive psychiatric services: results from a cluster randomized controlled trial on cognitive adaptation training delivered by nurses. *Schizophr Bull* 2020;46:1259-1268.

Thomas ML, Bismark AW, Joshi YB, et al. Targeted cognitive training improves auditory and verbal outcomes among treatment refractory schizophrenia patients mandated to residential care. *Schizophr Res* 2018;202:378-384.

Treichler EB, Thomas ML, Bismark AW, et al. Divergence of subjective and performance-based cognitive gains following cognitive training in schizophrenia. *Schizophr Res* 2019;210:215-220.

Van der Gaag M, Stant AD, Wolters KJ, Buskens E, Wiersma D. Cognitive–behavioural therapy for persistent and recurrent psychosis in people with schizophrenia-spectrum disorder: cost-effectiveness analysis. *Br J Psychiatry* 2011;198:59-65.

Van Oosterhout B, Krabbendam L, De Boer K, et al. Metacognitive group training for schizophrenia spectrum patients with delusions: a randomized controlled trial. *Psychol Med* 2014;44:3025-3035.

Vaskinn A, Løvgren A, Egeland MK, et al. A randomized controlled trial of training of affect recognition (TAR) in schizophrenia shows lasting effects for theory of mind. *Eur Arch Psychiatry Clin Neurosci* 2019;269:611-620.

Velligan DI, Tai S, Roberts DL, et al. A randomized controlled trial comparing cognitive behavior therapy, cognitive adaptation training, their combination and treatment as usual in chronic schizophrenia. *Schizophr Bull* 2015;41:597-603.

Vidarsdottir OG, Roberts DL, Twamley EW, Gudmundsdottir B, Sigurdsson E, Magnusdottir BB. Integrative cognitive remediation for early psychosis: results from a randomized controlled trial. *Psychiatry Res* 2019;273:690-8.

Vita A, De Peri L, Barlati S, et al. Psychopathologic, neuropsychological and functional outcome measures during cognitive rehabilitation in schizophrenia: a prospective controlled study in a real-world setting. *Eur Psychiatry* 2011;26:276-283.

Vita A, De Peri L, Barlati S, et al. Effectiveness of different modalities of cognitive remediation on symptomatological, neuropsychological, and functional outcome domains in schizophrenia: a prospective study in a real-world setting. *Schizophr Res* 2011;133:223-231.

Waller H, Emsley R, Freeman D, et al. Thinking Well: A randomised controlled feasibility study of a new CBT therapy targeting reasoning biases in people with distressing persecutory delusional beliefs. *J Behav Ther Exp Psychiatr* 2015;48:82-89.

Waller H, Landau S, Fornells-Ambrojo M, et al. Improving implementation of evidence based practice for people with psychosis through training the wider workforce: results of the GOALS feasibility randomised controlled trial. *J Behav Ther Exp Psychiatr* 2018;59:121-128.

Wang LQ, Chien WT, Yip LK, Karatzias T. A randomized controlled trial of a mindfulness-based intervention program for people with schizophrenia: 6-month follow-up. *Neuropsychiatr Dis Treat* 2016;12:3097-3110.

Westermann S, Rüegg N, Lüdtke T, Moritz S, Berger T. Internet-based self-help for psychosis: Findings from a randomized controlled trial. *J Consul Clin Psychol* 2020;88:937-950.

White R, Gumley A, McTaggart J, et al. A feasibility study of Acceptance and Commitment Therapy for emotional dysfunction following psychosis. *Behav Res Ther* 2011;49:901-907.

Wojtalik JA, Mesholam-Gately RI, Hogarty SS, et al. Confirmatory efficacy of cognitive enhancement therapy for early schizophrenia: results from a multisite randomized trial. *Psychiatr Serv* 2022;73:501-509.

Zhao W, Law S, Luo X, et al. First adaptation of a family-based ACT model in Mainland China: a pilot project. *Psychiatr Serv* 2015;66:438-441.

Zhu X, Song H, Chang R, et al. Combining compensatory cognitive training and medication self-management skills training, in inpatients with schizophrenia: A three-arm parallel, single-blind, randomized controlled trial. *Gen Hosp Psychiatry* 2021;69:94-103.

**Study characteristics**

The study characteristics for all trials included within the full review. All trials compared a psychological intervention to treatment as usual, and therefore comparator type was excluded from the table. Likewise, no trial measured hospitalization, and therefore this was excluded from the table also. For ease, the study characteristics are divided into two tables; the first presenting sample characteristics, intervention details and primary outcomes, and the second presenting secondary outcomes.

| Citation | Sample Size | Sample diagnoses | Sample substance use | Mean age | Proportion (%) males | Intervention duration | Latest follow-up | Psychosis outcomes | Psychosis findings |
| --- | --- | --- | --- | --- | --- | --- | --- | --- | --- |
| PE | | | | | | | | | |
| Aho-Mustonen et al (2011) | 39 | 100% SCZ or SCZA | 55.3% substance abuse | 40.7 | 90.7% | 8 weeks | 3 months | BPRS total score | No sig. difference between groups in BPRS change from baseline to follow-up (*t*=NR, *d*=NR, *p*=.76). |
| MCT | | | | | | | | | |
| Favrod et al (2014) | 52 | 82.7% SCZ, 17.3% SCZA | 40.4% AUD, 15.4% CUD | 50.1 | 68.4% | 8 weeks | 6 months | PSYRATS: delusions and hallucinations.  PANSS positive. | At follow-up, sig. lower PSYRATS delusions (*F*(1,47) = 4.70, *p*=.04, *d*=0.64), hallucinations (*F(*1,32) = 4.46, *p=*.04, *d=*0.61), and PANSS positive symptoms score (*F*(1,47) = 4.95, *p*=.03, *d*=0.48) for intervention group than control when controlling for baseline scores. |
| Kuokkanen et al (2014) | 20 | 100% SCZ | 55% substance abuse | 43.56 | 100% | 4 weeks | 6 months | PSYRATS delusions, and PANSS items P1, P6, and G12 (summated). | Sig. greater change in PANSS (*W*=19.35, *p*<.001) at follow up, but no significant difference in change in PSYRATS delusions (*W*=4.87, *p*>.05). |
| Moritz et al (2011) | 36 | 100% SCZ spectrum disorders (disorders and % NR) | 53% DSM substance abuse | 32.75 | 77.8% | 8 weeks | Immediately post intervention | PSYRATS delusions and hallucinations, and PANSS: positive, negative, disorganization, excitement, distress, and total. | No sig. differences for any of the PANSS subscale change scores, *t*(34)<1.11, *p*>.2, *d*<.38, or PSYRATS delusion or hallucination subscales (*d*=NR, *p*>.05). |
| CBT | | | | | | | | | |
| Gleeson et al (2013) | 81 | 33.3% SCZ, 11.1% SCZP, 4.9% SCZA, 1.2% DD, 3.7% SI-PD | Abuse/ dependence:  24.7% alcohol, 51.9% cannabis, 7.4% opioid, 3.7% cocaine, 14.8% hallucinogen, 18.5% amphetamine | 20.1 | 63.8% | 7 months | 30 months | BPRS positive and total, and SANS: affect, alogia, avolition, anhedonia, and attention. | No sig. group x time interaction for BPRS total score (*F*(5,122.6) = 1.13, *p*=.35), BPRS positive symptoms (*F*(5,148.5) = .41, *p*=.84), SANS affect (*F*(5,130.9) = 1.14, *p*=.34), SANS avolition (*F*(5,148.1) = 1.70, *p*=.14), or SANS anhedonia (*F*(5,144.6) = 1.50, *p*=.19). For all scales, ES=NR.  Sig. group x time interaction for SANS alogia (*F*(5,214.1 = 4.15, *p*=.001), SANS attention (*F*(5,154.2 = 2.71, *p*=.023), and SANS summary scores (*F*(5,185.5 = 2.28, *p*=.049), with the TAU group reporting greater improvements than the intervention for all subscales (*p*=.006, *p*=.001, *p*=.018, for endpoint analysis, respectively). For all scales, ES=NR. |
| Khazaal et al (2015) | 172 | 81.4% SCZ, 18.6% other psychotic disorders | 15.6% SUD | 37.1 | 62.2% | 3 months | 9 months | BPRS: negative, positive, affect, resistance, activation. | No sig. difference between scores for any BPRS subscale at follow-up when controlling for baseline scores (*F* =NR, *ES*=NR, all *p*>.05). |
| OPUS | | | | | | | | | |
| Secher et al (2015) | 347 | 66.2% SCZ, 14.4% SCZT, 4.6% DD, 4.6% SCZA | 27% substance abuse diagnosis | 26.6 | 59.1% | 2 years | 10 years | Combined SAPS and SANS: psychotic, negative, disorganized | No sig. difference between groups for psychotic (EMD = 0.02, *p*=.91), negative (EMD = 0.10, *p*=.36) or disorganized (EMD = -0.02, *p*=.79) scales at follow-up. |

Where mixed samples are investigated, only the % of disorders of interest are reported within the sample diagnoses. RCT = randomised controlled trial, SCZ = schizophrenia, SCZA = schizoaffective disorder, SCZP = schizophreniform disorder, DD = delusional disorder, SI-PD = substance-induced psychotic disorder, SCZT = schizotypal disorder, AUDIT = Alcohol Use Disorder Identification Test, AUD = Alcohol Use Disorder, CUD = Cannabis Use Disorder, NR = not reported, sig. = significant, BPRS = Brief Psychiatric Rating Scale, PANSS = Positive and Negative Syndrome Scale, SANS = Schedule for the Assessment of Negative Symptoms, SAPS = Schedule for the Assessment of Positive Symptoms, PE = psychoeducation, MCT = metacognitive training, CBT = cognitive behavioral therapy, OPUS = specialized assertive early intervention program. ES = effect size, EMD = estimated mean difference.

| Citation | Substance use measure | Substance use findings | Quality of life measure | Quality of life findings | General functioning measure | General functioning findings | Relapse measure | Relapse findings | Other adverse effects or treatment failures |
| --- | --- | --- | --- | --- | --- | --- | --- | --- | --- |
| PE | | | | | | | | | |
| Aho-Mustonen et al (2011) |  |  | 15D Sintonen instrument | No. sig difference in quality of life at follow-up (*t*=NR, *p*=.06, *d*=0.62) |  |  |  |  | Sig. greater increase in irritability for intervention group compared to TAU (*t*=.2.12, *p*=.04, *d*=-0.69). |
| MCT | | | | | | | | | |
| Favrod et al (2014) |  |  |  |  |  |  |  |  | None reported. |
| Kuokkanen et al (2014) |  |  |  |  |  |  |  |  | None reported. |
| Moritz et al (2011) |  |  | WHOQOLBRIEF: physical health, psychological health, social relationships, and environment subscales. | Sig. greater improvement in social relationships for intervention group compared to TAU (*t*(32) = 2.23, *p*=.03, *d*=.77). No sig. difference in change on any other subscale (all *t*<.81, *p>*.4, *d*<.28). |  |  |  |  | None reported. |
| CBT | | | | | | | | | |
| Khazaal et al (2015) |  |  |  |  | GAF and SOFAS | No. sig difference between groups at follow-up on either measure (both *p*>.05, *F*=NR, ES=NR). |  |  | None reported. |
| Gleeson et al (2013) | ASSIST: alcohol and cannabis, and AUDIT and SDS | No sig. group x time interaction for AUDIT (*F*(5,130.1) = .73, *p*=.60), SDS (*F*(5,118.3) = .6, *p*=.70), ASSIST alcohol (*F*(5.145.1) = 1.47, *p*=.20) or ASSIST cannabis (*F*(5,140.9) = .33, *p*=.90). For all measures, ES=NR. | WHOQOLBRIEF: physical health, psychological health, social relationships, and environment subscales. | No sig. group x time interaction on the physical health (*F*(5,135.9) = .63, *p*=.68), psychological health (*F*(5,124.5) = .81, *p*=.55), social relationships (*F*(5,142.4) = 1.16, *p*=.33) or environmental subscales (*F*(5,138.1) = .56, *p*=.73). For all measures, ES=NR. | SOFAS | Sig. group x time interaction (*F*(5,128.4) = 2.30, *p*=.049, ES=NR), with the intervention group reporting sig. lower scores at follow-up than TAU (*p*=.039). | Increases from ≤3 to ≥6 on any of 3 BPRS items within 1 week OR increase from ≤3 (for at least 1 month) on all 3 items followed by a score of 5 on any of the 3 items plus a 2-point increase on one of the other scales (within 1 week) or a rating of 5 on any one of the 3 scales for at least 1 month, OR ≥2 increase in 1 item to a rating ≥6 or a 1-point rise to a rating ≥6 with a 2 point rise on one of the other 2 items (for at least 1 week). | No. sig differences in relapse rates at follow-up (*χ^2^*(1) = 1.15, *p*=.28). |  |
| OPUS | | | | | | | | | |
| Secher et al (2015) |  |  |  |  | GAF: symptom and functioning subscales | No sig. group x time interaction for either symptom (EMD = -1.22, *p*=.48) or functioning subscales (EMD = -.76, *p*=.65). |  |  | Sig. more participants in the intervention group spent a day in a homeless shelter than the TAU group, during the intervention. |

*Note:* sig. = significant, SQLS-R4 = Schizophrenia Quality of Life Scale Revision 4, WHOQOLBRIEF = Brief Quality of Life Questionnaire of the World Health Organization, GAF = Global Assessment of Functioning, SOFAS = Social and Occupational Functioning Scale, ASSIST = Alcohol, Smoking, and Substance Involvement Screening Test, AUDIT = Alcohol Use Disorder Identification Test, SDS = Severity of Dependence Scale, PE = psychoeducation, MCT = metacognitive training, CBT = cognitive behavioral therapy, OPUS = specialized assertive early intervention program, ES = effect size.
